# Effects of Different Basal Cell Culture Media upon the Osteogenic Response of hMSCs Evaluated by ^99m^Tc-HDP Labeling

**DOI:** 10.3390/ijms23116288

**Published:** 2022-06-03

**Authors:** Tobias Grossner, Uwe Haberkorn, Jakob Hofmann, Tobias Gotterbarm

**Affiliations:** 1 Clinic for Orthopedics and Trauma Surgery, Center for Orthopedics, Trauma Surgery and Paraplegiology, University Hospital Heidelberg, 69120 Heidelberg, Germany; jakob.hofmann@med.uni-heidelberg.de; 2 Department of Nuclear Medicine, University Hospital Heidelberg, 69120 Heidelberg, Germany; uwe.haberkorn@med.uni-heidelberg.de; 3 Clinical Cooperation Unit Nuclear Medicine, German Cancer Research Center (DKFZ), 69120 Heidelberg, Germany; 4 Translational Lung Research Center Heidelberg (TLRC), German Center for Lung Research (DZL), 69120 Heidelberg, Germany; 5 Department of Orthopedics and Traumatology, Kepler University Hospital, 4020 Linz, Austria; tobias.gotterbarm@kepleruniklinikum.at

**Keywords:** mesenchymal stem cells, osteogenic differentiation, basal cell culture media, DMEM, ^99m^Tc-HDP labeling, hydroxyapatite, alizarin red staining, α-MEM

## Abstract

The osteogenic differentiation of mesenchymal stem cells is now a standard procedure in modern bone tissue engineering. As this is a promising field for future clinical applications, many cell culture media exist to promote osteogenic differentiation. Prior to differentiation, cells must be expanded to obtain sufficient numbers for experiments. Little evidence is available regarding the optimal media combination for expansion and differentiation to maximize the osteogenic response. Therefore, human BM-MSCs (*n* = 6) were expanded in parallel in DMEM (Dulbecco’s Modified Eagle Medium) LG (Low Glucose) and α-MEM (Minimum Essential Media alpha-modification), followed by simultaneous monolayer differentiation toward the osteogenic lineage in: 1. DMEM LG (Low Glucose), 2. DMEM HG (High Glucose), 3. α-MEM, 4. “Bernese medium”, and 5. “Verfaillie medium”, with a corresponding negative control (total 20 groups). As a marker for osteogenic differentiation, hydroxyapatite was accessed using radioactive ^99m^Tc-HDP labeling and quantitative alizarin red staining. The results indicate that all media except “Bernese medium” are suitable for osteogenic differentiation, while there was evidence that DMEM LG is partly superior when used for expansion and differentiation of BM-hMSCs. Using “Verfaillie medium” after DMEM LG expansion led to the highest grade of osteogenic differentiation. Nevertheless, the difference was not significant. Therefore, we recommend using DMEM LG for robust osteogenic differentiation, as it is highly suitable for that purpose, economical compared to other media, and requires little preparation time.

## 1. Introduction

Osteogenic differentiation of human bone marrow-derived mesenchymal stromal cells (MSCs, also referred to as mesenchymal stem cells) is a standard procedure in modern bone tissue engineering, and was established in the late 1990s [1]. There is a high demand for innovative osteoregenerative therapies due to the fast-growing number of critical-size bone defects and non-unions in an aging population [2]. These pathologies are usually treated by autologous bone transplantation at the expense of a significant donor site morbidity [3,4,5,6]. Therefore, it is no surprise that researchers worldwide are investigating stem cell-based treatment options [2,7,8]. While there have been promising attempts to apply MSCs in these conditions, no breakthrough has been made [9,10]. This might be because the biochemical interactions of MSCs in relation to their osteogenic potential in vitro and in vivo are still not fully understood [9]. Many different methods exist to induce the differentiation of these cells toward the osteogenic lineage in vitro. In addition, various attempts have been made to enhance the osteogenic potential of stem cells by using different osteogenic supplements besides the standard supplements dexamethasone, ascorbic acid, and ß-glycerol phosphate [11]. Vitamin D, bone-morphogenic proteins, other chemical inducers, and physical stimuli are the focus of the latest research [12,13,14,15].

Surprisingly, less attention is paid to the basal cell culture media—especially in terms of the media used for cell expansion prior to the osteogenic differentiation as well as the cell culture media used for the differentiation of the cells—although it is well known that the cell culture medium has an essential impact on the level of osteogenic differentiation of MSCs. The osteogenic cellular response, reflected in the amount of deposited hydroxyapatite, can therefore significantly differ depending on the media used within the specific stages (cell expansion and cell differentiation) of the cell culture [16,17,18]. As there is increasing evidence for the importance of proper cell culture media, some researchers have already revealed significant insights into how cell culture media affect osteogenic and chondrogenic differentiation of MSCs [17,19,20,21].

As the choice of cell culture media can result in significant differences in the osteogenic cellular response, the media must be carefully chosen to avoid an unwanted impact on an experimental design/leading question of an experiment, especially when the cell culture media is not the focus of the experiment at all (e.g., evaluation of the influence of different surface conditions of scaffolds toward the osteogenic response). Therefore, for a correct interpretation of experimental results in the field of osteogenic tissue engineering, it is very important for researchers to have an extensive and specific knowledge of which media affect the osteogenic differentiation at what stage to better classify and sort results and to be able to draw correct conclusions in the future.

Dulbecco’s Modified Eagle Medium (DMEM) is one of the most frequently used basal cell culture media, as it works efficiently with mammalian cell lines such as mesenchymal stem cells. It is also suitable for differentiating MSCs toward the osteogenic lineage [1]. The original composition contains 1 g/L glucose and is commonly called DMEM low glucose. Recently, a modification of the formulation was made which is referred to as DMEM high glucose, containing 4.5 g/L glucose [22,23]. As DMEM contains no proteins, growth factors, or lipids, a proper source for these supplements needs to be added, such as fetal calf serum.

Another popular basal cell culture medium for cell culture in tissue engineering is Minimum Essential Medium alpha-modification, also referred to as α-MEM. This medium is highly suitable for the osteogenic differentiation of human bone marrow cells as, due to the α-modification, it now contains non-essential amino acids, Vitamin B_12_, biotin, and ascorbic acid [24,25,26].

Using Dulbecco’s Modified Eagle Medium high glucose in a 1:1 mixture with Ham’s F-12 Nutrient mixture + L-Glutamine leads to a medium called DMEM/F12. Due to its high glucose level, amino acids, vitamins, and the F12 mix containing zinc, putrescin, hypoxanthine, and thymidine, it is highly suitable for primary cell culture. TGF-ß (Transforming Growth Factor) and FGF-2 (Fibroblast Growth Factor-2) may be optionally added to DMEM/F12, leading to a more elaborate cell culture medium known as “Bernese medium” that favors chondrogenic differentiation of MSCs [27,28].

By adding MCDB (Keratinocyte Basal Medium), ITS (Insulin-Transferrin-Selenium) supplement, dexamethasone, ascorbic acid, PDGF (Platelet-Derived Growth Factor), and EGF (Epidermal Growth Factor) to DMEM high glucose, a variation of the “Verfaillie medium” is created, which enhances cell proliferation and boosts chondrogenic proliferation and osteogenic differentiation of mesenchymal stem cells [29]. Like the “Bernese medium”, this medium is a complex medium, and so it is not referred to as a basal cell culture medium.

For bone tissue engineering (BTE), a certain number of cells are needed to perform in vitro experiments. Due to individual donor site anatomy (e.g., femoral cave, iliac crest), there is a natural limit to the total number of cells that can be harvested. Therefore, the cells must be expanded in vitro prior to the actual experiment to obtain sufficient numbers. Here, too, different cell culture media are used, often without knowing whether these media might already have a positive or negative impact on the desired differentiation, or if there is an optimal combination between the cell culture media used for expansion and the cell culture media used for differentiation.

**Figure 1:**
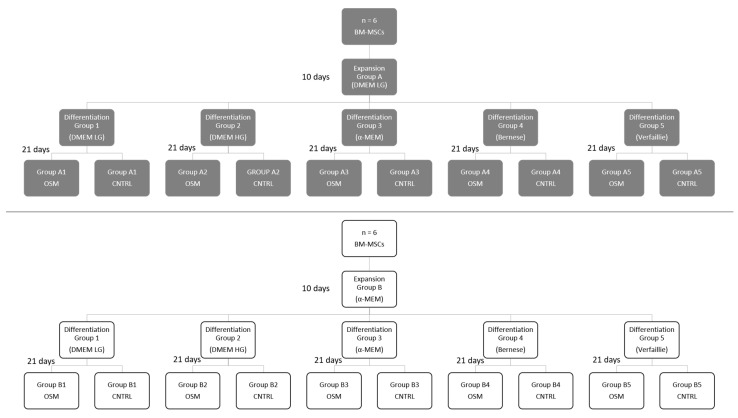
Overview of all 20 groups used in this study. Cell expansion in group A or group B for a duration of 10 days, followed by subsequent differentiation in one of the five differentiation media for 21 days in OSM and CNTRL. OSM = osteogenic media, CNTRL = control group.

This study was performed to determine the optimal basal cell culture media used for the expansion period (DMEM low glucose or α-MEM) as well as for the subsequent differentiation period (DMEM low glucose, DMEM high glucose, α-MEM, “Bernese medium”, or “Verfaillie medium”) in order to maximize the osteogenic differentiation response of bone marrow mesenchymal stem cells, determined by the amount of synthesized hydroxyapatite. Figure 1 gives an overview of the study and the groups used in the experiment.

In BTE bone regeneration, the synthesis of a mineralizing matrix is vitally important. Therefore, the amount of hydroxyapatite produced was used as the marker to determine the grade of osteogenic differentiation within this study. To directly access the amount of mineral in vitro, the well-established methods von Kossa staining and quantitative alizarin red staining are usually used [30,31]. Recently, ^99m^Tc-HDP labeling has been employed as a novel, non-destructive, and highly sensitive method to access the hydroxyapatite content in monolayer and 3D cultures of human MSCs [32,33]. The method is very accurate and, just as in quantitative alizarin red staining, the mineralized matrix remains intact so that the hydroxyapatite content can be investigated [34]. The method correlates well with common methods for osteogenic quantification such as access of alkaline phosphatase activity and immunohistochemical staining [35].

## 2. Results

### 2.1. FACS and Cell Expansion Analysis—Comparison of Cell Expansion in DMEM and a-MEM

The phenotype analysis of the harvested cells confirmed the presence of a highly purified population of mesenchymal stromal cells, fulfilling the phenotype requirements as stated by the International Society for Cellular Therapy (≥95%+: CD 73, CD 90, CD 105; ≤2%+: CD 14, CD 19, CD 34, CD 45) [36].

Initially, to normalize the cell numbers in the expansion phase of the experiment for all donors, 250,000 cells from each donor were thawed and seeded into T-150 flasks. After the standardized cell expansion period of 10 days the mean cell count (*n* = 6) for the cells expanded in DMEM LG (expansion group A) was 1,870,833 cells, while the mean cell count for the cells expanded in α-MEM (expansion group B) was 1,958,333 cells. (Please see Section 4.2 and Section 4.3 in Materials and Methods for the full description of cell harvest and expansion).

**Figure 2:**
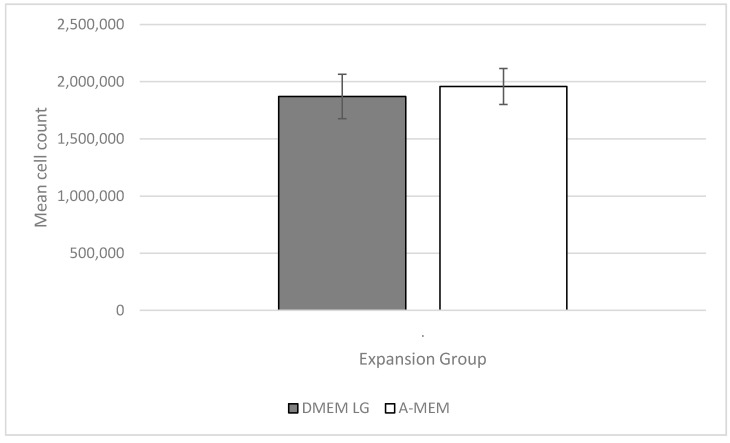
Mean cell count (*n* = 6) for the cells expanded in DMEM LG (expansion group A) and expanded in α-MEM (expansion group B) +/− SD. No significance between the two groups.

A Kolmogorov–Smirnov Test of the cell counts revealed a normal distribution for the individual numbers of both groups, while a subsequent Student’s *t*-test showed no statistical difference between the two expansion media in regard to the cell expansion (*p* ≥ 0.05) (Figure 2).

### 2.2. Descriptive Statistics for ^99m^Tc-HDP Labeling and Alizarin Red Staining—Comparison of ^99m^Tc-HPD Labeling and Alizarin Red Staining as a Means to Assess Differentiation of Cells Cultured in Different Media

When the amount of hydroxyapatite from the osteogenic-induced cell cultures (OSM groups) was accessed by ^99m^Tc-HDP labeling, the highest uptake of the tracer was seen in group A5 (expansion group A DMEM low glucose/differentiation group 5 “Verfaillie medium”) with 47,328 counts/180 s followed by B5 (expansion group B α-MEM/differentiation group 5 “Verfaillie medium”) with 39,670 counts/180 s, and A1 (expansion group A DMEM low glucose/differentiation group 1 DMEM low glucose) with 39,356 counts/180 s. Distinctly behind these results were the uptake rates for group A2 (expansion group A DMEM low glucose/differentiation group 2 DMEM high glucose) with 30,847 counts/180 s, A3 (expansion group A DMEM low glucose/differentiation group 3 α-MEM) with 29,948 counts/180 s, and B3 (expansion group B α-MEM/differentiation group 3 α-MEM) with 29,373 counts/180 s, followed by group B1 (expansion group B α-MEM/differentiation group 1 DMEM low glucose) with 21,573 counts/180 s and B2 (expansion group B α-MEM/differentiation group 2 DMEM high glucose) with 17,382 counts/180 s. The lowest uptake results were seen for group A4 (expansion group A DMEM low glucose/differentiation group 4 “Bernese medium”) and B4 (expansion group B α-MEM/control group 4 “Bernese medium”) with only 2026 counts/180 s and 2167 counts/180 s, respectively.

Alizarin red analysis of the cell cultures revealed very similar results. Here, too, group B5 (expansion group B α-MEM/differentiation group 5 “Verfaillie medium”) had the highest concentration of the stain (4.55 µg/mL), followed by group A5 (expansion group A DMEM low glucose/differentiation group 5 “Verfaillie medium”) with 4.54 µg/mL, and A3 (expansion group A DMEM low glucose/differentiation group 3 α-MEM) with 4.36 µg/mL. The lowest concentrations were found, similar to the prior results, for group A4 (expansion group A DMEM low glucose/differentiation Group 4 “Bernese medium”) with 1.91 µg/mL, and B4 (expansion group B α-MEM/control group 4 “Bernese medium”) with 0.87 µg/mL.

Within the non-osteogenic negative control groups (CNTRL), the uptake values for the ^99m^Tc-HDP labeling were much lower than the results seen within the osteogenic groups, except for the osteogenic groups A4 (expansion group A DMEM low glucose/differentiation group 4 “Bernese medium”) and B4 (expansion group B α-MEM/control group 4 “Bernese medium”). Their results were similar to the results of the negative control groups. However, the highest uptake was seen for group A2 (expansion group A DMEM low glucose/differentiation group 2 DMEM high glucose) with 4981 counts/180 s, while the lowest uptake was measured for B1 (expansion group B α-MEM/differentiation group 1 DMEM low glucose) with 2575 counts/180 s.

**Figure 3:**
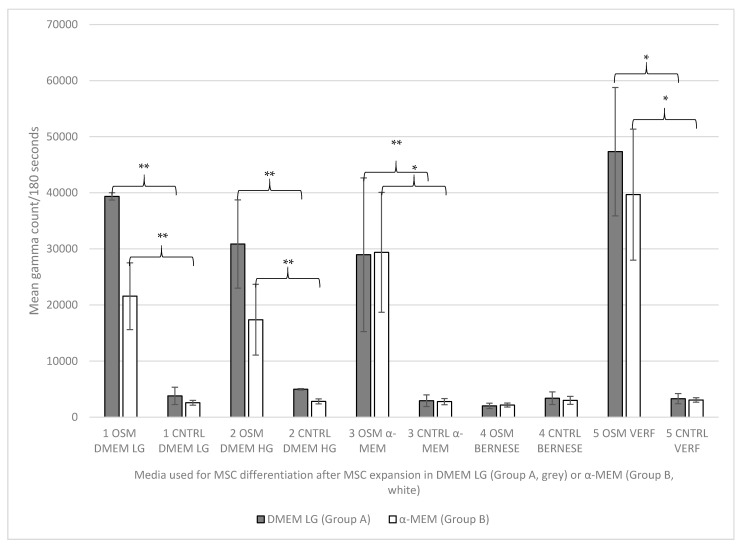
Descriptive statistics for the tracer uptake (gamma count mean values +/− SD) for all groups. The grey bars reflect the use of expansion media A (DMEM LG) and the white bars reflect the use of expansion media B (α-MEM) prior to differentiation, according to the designated group (1–5). OSM = osteogenic media, CNTRL = negative control group. Significance: ** = *p* ≤ 0.01; * = *p* ≤ 0.05.

**Figure 4:**
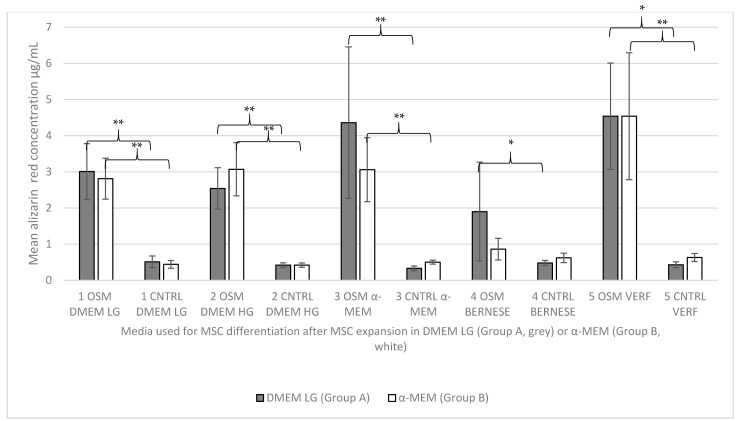
Descriptive statistics for the alizarin red concentration in µg/mL (mean values +/− SD) for all groups. The grey bars reflect the use of expansion media A (DMEM LG) and the white bars reflect the use of expansion media B (α-MEM) prior to differentiation, according to the designated group (1–5). OSM = osteogenic media, CNTRL = negative control group. Significance: ** = *p* ≤ 0.01; * = *p* ≤ 0.05.

The corresponding results are shown in Figure 3 and Figure 4.

### 2.3. Initial Assessment of All Data for Normal Distribution Using the Kolmogorov–Smirnov Test

#### 2.3.1. ^99m^Tc-HDP Labeling

The test for normal distribution of the values of the gamma counts, acquired over 180 s (*n* = 120) using the ^99m^Tc-HDP labeling method, revealed that the results were normally distributed with an asymptotic 2-sided significance of *p* ≤ 0.001 and a 2-sided Monte Carlo significance of *p* ≤ 0.001.

#### 2.3.2. Alizarin Red Staining

The test for normal distribution of the values of the alizarin red staining (*n* = 120) revealed that the results were normally distributed as well with an asymptotic 2-sided significance of *p* ≤ 0.001 and a 2-sided Monte Carlo significance of *p* ≤ 0.001.

### 2.4. Pre-Test of the Data Using the Kruskal–Wallis Test to Determine Global Significances before Further Detailed Statistical Assessment

#### 2.4.1. ^99m^Tc-HDP Labeling

This global test for significances between the groups revealed a Kruskal–Wallis H of 78,545, with a highly significant asymptotic significance of *p* ≤ 0.001. Hence, the data was further analyzed using the Mann–Whitney U test due to the sample size of each group (*n* = 6).

#### 2.4.2. Alizarin Red Staining

This global test for significances between the groups revealed a Kruskal–Wallis H of 80,969, with a highly significant asymptotic significance of *p* ≤ 0.001. Therefore, the data were further analyzed using the Mann-Whitney U test due to the sample size of each group (*n* = 6).

### 2.5. Mann–Whitney U Test to Reveal Statistical Significances between the Osteogenic and Control Groups

#### 2.5.1. Paired Mann–Whitney U Test for All Osteogenic Groups (OSM) and the Corresponding Negative Control Group (CNTRL)

##### ^99m^Tc-HDP Labeling

**Figure 5:**
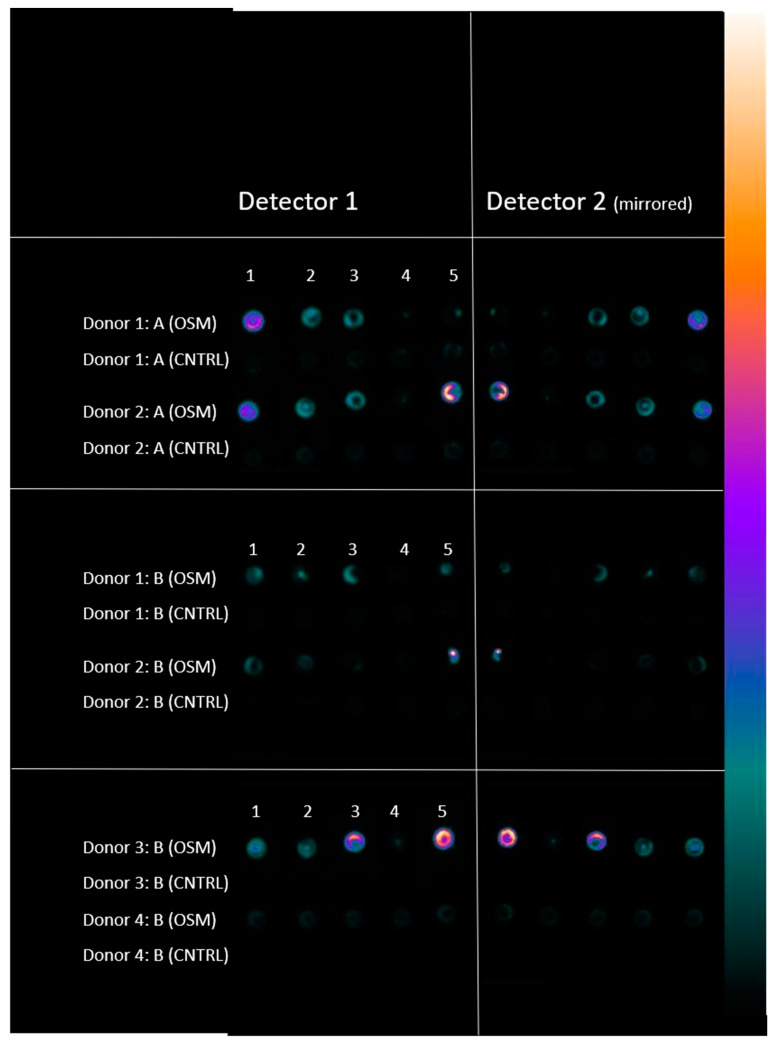
Gamma camera live image of the cell cultures during the acquisition of the tracer uptake after 180 s for Donors 1 and 2, expanded in DMEM LG (A), followed by differentiation in specific media according to the individual group (1–5) and for donors 1, 2, 3, and 4, expanded in α-MEM (B), followed by differentiation in specific media according to the individual group (1–5). OSM = osteogenic media, CNTRL = negative control group. Counts were acquired using detector 1, while detector 2 always displayed a mirrored image which was not analyzed.

The test revealed a significant positive influence for all osteogenic media compared to their corresponding negative control groups in terms of osteogenic differentiation, except for group A4 (expansion DMEM LG/differentiation Bernese medium) (*p* = 0.132) and group B4 (expansion α-MEM/differentiation Bernese medium) (*p* = 0.310). These results show that no significant higher uptake of the tracer occurred within these two particular osteogenic groups (A4 and B4) in direct comparison to their negative control group, which shows that these two media combinations did not lead to a significant deposition of hydroxyapatite that would reflect osteogenic differentiation. Table 1 shows all results of the paired analysis, and Figure 5 shows the overview gamma camera analyzation image acquired for donors 1 and 2. Similar images were produced for the remaining donors.

##### Alizarin Red Staining

**Figure 6:**
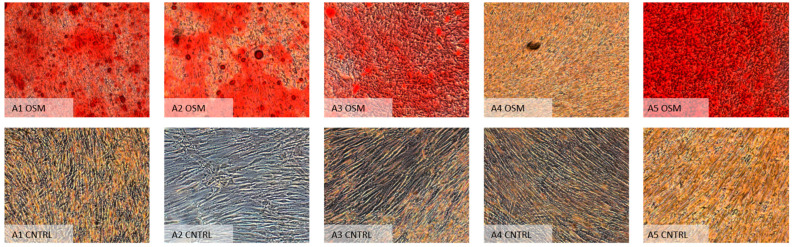
Microscopic images (10×) of all groups from one donor expanded in DMEM LG (condition A), followed by differentiation in specific media according to the individual group (1–5). OSM = osteogenic media, CNTRL = negative control group.

**Figure 7:**
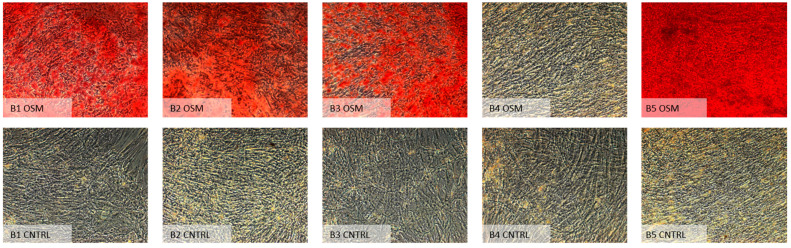
Microscopic images (10×) of all groups from one donor expanded in α-MEM (condition B), followed by differentiation in specific media according to the individual group (1–5). OSM = osteogenic media, CNTRL = negative control group.

Similar to the analysis of the paired Mann–Whitney U test results for the ^99m^Tc-HDP labeling, the analysis of the alizarin red staining results likewise revealed a significantly higher uptake of the stain within all osteogenic groups except for group B4 (expansion α-MEM/differentiation Bernese medium) (*p* = 1.0). Contrary to the previous results from the ^99m^Tc-HDP labeling, the test for group A4 (expansion DMEM LG/differentiation “Bernese medium”) showed a significant difference (*p* = 0.026). Table 3 shows all results of the paired analysis, while Figure 6 and Figure 7 show microscopic overviews of the Alizarin Red-stained cultures of all the different groups.

#### 2.5.2. Mann–Whitney U Test to Determine Significant Differences between All Osteogenic Groups (OSM) where Group A1 OSM (DMEM LG/DMEM LG) Is Defined as the Primary Reference Group/Gold Standard 

This test was performed to reveal whether there was a superior group regarding osteogenic differentiation, compared to the group A1 OSM (DMEM LG/DMEM LG), which was defined as the primary standard.

The osteogenic differentiation of mesenchymal stem cells in DMEM LG, with the expansion of the cells using DMEM LG as well, led to a robust osteogenic differentiation. The analysis of the cell culture showed high uptake values of the tracer for ^99m^Tc-HDP labeling (39,356 counts/180 s) as well as for quantitative alizarin red staining (3.01 µg/mL).

There was no significant difference to the uptake values for the groups A2 (expansion group A DMEM low glucose/differentiation group 2 DMEM high glucose), A3 (expansion group A DMEM low glucose/differentiation group 3 α-MEM), and A5 (expansion group A DMEM low glucose/differentiation group 5 “Verfaillie medium”); nor for the groups B3 (expansion group B α-MEM/differentiation group 3 α-MEM) and B5 (expansion group B α-MEM/differentiation group 5 “Verfaillie medium”). This indicates that no significantly higher or lower osteogenic differentiation was achieved in these groups by using expansion and differentiation media other than the reference media.

However, there was a significantly lower uptake for the radioactive tracer in the osteogenic group A4 (expansion group A DMEM low glucose/differentiation group 4 “Bernese medium”) that was previously expanded with DMEM LG, while three of the groups, expanded with α-MEM followed by expansion with B1 (expansion group B α-MEM/differentiation group 1 DMEM low glucose), B2 (expansion group B α-MEM/differentiation group 2 DMEM high glucose), and B4 (expansion group B α-MEM/differentiation group 4 “Bernese medium”) all showed a significantly lower uptake of the tracer. This indicates that the individual osteogenic response of the cells was far less than in the reference group, probably due to the chosen media combination.

Surprisingly, the analysis of the alizarin red quantification revealed a significant difference for only one group. The concentration of the stain was significantly lower in group B4 (expansion group B α-MEM/differentiation group 4 “Bernese medium”) compared to the reference group, while the comparison of all other groups showed no significantly lower uptake of the stain and, therefore, no significantly lower amount of hydroxyapatite deposited (Table 2).

#### 2.5.3. Mann–Whitney U Test for All Osteogenic Groups (OSM) with Group B3 OSM (α-MEM/α-MEM) Defined as the Reference Group 

This test was performed to reveal whether there was a superior group regarding osteogenic differentiation, with the group A1 OSM (DMEM LG/DMEM LG) defined as secondary standard.

The osteogenic differentiation of mesenchymal stem cells in α-MEM with the expansion of the cells also using α-MEM led to a robust osteogenic differentiation as well. The cell culture analysis showed high uptake values of the tracer for ^99m^Tc-HDP labeling (29,373 counts/180 s) and quantitative alizarin red staining (3.06 µg/mL).

There was no significant difference in the uptake values for the groups A2 (expansion group A DMEM low glucose/differentiation group 2 DMEM high glucose), A3 (expansion group A DMEM low glucose/differentiation group 3 α-MEM), and A5 (expansion group A DMEM low glucose/differentiation group 5 “Verfaillie medium”), nor for the groups B3 (expansion group B α-MEM/differentiation group 3 α-MEM) and B5 (expansion group α-MEM/differentiation group “Verfaillie medium”). This indicates that no significantly higher or lower osteogenic differentiation was achieved in these groups by using expansion and differentiation media other than the reference media.

However, there was a significantly lower uptake for the radioactive tracer in the osteogenic groups that were expanded with A4 (expansion group A DMEM low glucose/control group 4 “Bernese medium”), while three of the groups expanded with a-MEM followed by expansion with B1 (expansion group B α-MEM/differentiation group 1 DMEM low glucose), B2 (expansion group B α-MEM/differentiation group 2 DMEM high glucose), and B4 (expansion group B α-MEM/differentiation group 4 “Bernese medium”) all showed a significantly lower uptake of the tracer. This indicated that the individual osteogenic response of the cells was far less than in the reference group due to the chosen media combination.

Surprisingly, the analysis of the alizarin red quantification revealed a significant difference for only one group. The concentration of the stain was significantly lower in group B4 (expansion group B α-MEM/differentiation group 4 “Bernese medium”) compared to the reference group, while for all other groups, the comparison revealed no significantly lower uptake of the stain and, therefore, no significantly lower amount of hydroxyapatite deposited (Table 3).

### 2.6. Mann–Whitney U Test for the Corresponding Pairs Regarding the Expansion Media

To verify any significant differences between the media used for expansion, pairs were formed of the groups where a similar media was used for differentiation while the expansion media was either α-MEM or DMEM LG. Here, only the comparison of group A1 (expansion DMEM LG/differentiation DMEM LG) with B1 (expansion α-MEM/differentiation DMEM LG) revealed a significant effect for the radioactive labeling method (*p* = 0.041), while the alizarin red staining showed no significant effect (*p* = 1.0). All other pairs showed no significant difference.

In addition, the mean values of all osteogenic groups where DMEM LG was used for expansion were compared to the mean values of all groups previously expanded using α-MEM. Here, the mean value for all groups where DMEM LG was used for expansion was 29,700 gamma counts/180 s and 3.27 µg/mL alizarin red stain, while for all groups where α-MEM was used, the mean values were 22,033 gamma counts/180 s and 2.87 µg/mL alizarin red stain. Even if the uptake and staining results showed higher results for the DMEM LG expansion groups, the Mann–Whitney U test revealed no significant difference between the results (*p* = 0.287 and *p* = 0.865, respectively).

### 2.7. Pearson’s Correlation ^99m^Tc-HDP Labeling vs. Alizarin Red Staining

The results were correlated with the results from the gold-standard alizarin red labeling (*n* = 120) to verify the accuracy of the novel method of ^99m^Tc-HDP labeling to access the amount of hydroxyapatite. The two methods had a highly significant correlation (2-sided significance *p* ≤ 0.001), and this was very robust with a Pearson’s correlation coefficient of r^2^ = 0.799.

## 3. Discussion

Osteogenic differentiation of mesenchymal stem cells has become a standard procedure in modern bone tissue engineering. It was first described in 1997, when the osteogenic supplements dexamethasone, ascorbic acid, and ß-glycerol phosphate were used [1]. Various modifications of the initial protocol have been tested using the same supplements in different concentrations, as well as other inducers known to favor osteogenic differentiation, such as members of the bone-morphogenic proteins (BMPs) and vitamin D [11,14,37]. Some of the alternative methods showed impressive results regarding the osteogenic response.

Nevertheless, the initial publication is still the reference standard to differentiate mesenchymal stem cells into the osteogenic lineage. This allows a thorough investigation of the osteogenic lineage but is usually done to prove the multilineage differentiation capacity of various cell lines [36]. It is known that various concentrations of the osteogenic supplement result in a different osteogenic response [11]. It has also been shown that a robust osteogenic differentiation can be achieved using different basal cell culture media such as DMEM and α-MEM, while the choice of differentiation media might affect the osteogenic outcome parameters. DMEM is known to promote mineralization of an extracellular matrix superior to α-MEM, whereas differentiation of MSCs using α-MEM will lead to a higher APase activity, which is also an established marker for the osteogenic response [26]. Even more complicated is the choice of media, because the type of expansion medium used determines the stem cell character and thus the differentiation potential [18].

There is a lack of test data concerning different combinations with the goal of maximizing osteogenic differentiation, defined by the synthesized hydroxyapatite content. Therefore, this study was performed to investigate different media for expansion and differentiation and define the most efficient combination to maximize the osteogenic response of mesenchymal stem cells while also focusing on economic parameters.

The general evaluation of the osteogenic response for all different groups/types of media, and, therefore, the suitability for use as osteogenic differentiation media, showed a proper osteogenic response for all groups (Table 3) except for the two groups in which the “Bernese medium” was used for differentiation. Group A4 OSM compared to group A4 CNTRL showed no adequate osteogenic response in terms of ^99m^Tc-HDP evaluation, while group B4 OSM compared to group B4 CNTRL showed no osteogenic response by ^99m^Tc-HDP evaluation or by alizarin red staining evaluation. This could be caused by the fact that the “Bernese medium” was devised to promote chondrogenic differentiation, while it apparently lacks the ability to support osteogenic differentiation [27]. As there is a mutual osteo-chondrogenic pathway during the differentiation of MSCs [38], it was theoretically assumed that combining the chondrogenic properties of the “Bernese medium” with osteogenic supplements might have a significant effect on osteogenesis. As this was not proven, we cannot recommend this medium for any experiment investigating osteogenesis. That the other media tested work quite well for osteogenic differentiation has already been shown elsewhere, and this knowledge is supported by our results [1,11,23,29].

As assumed and confirmed by our results, the two basal cell culture media (DMEM LG and α-MEM), defined within this experiment as first- and second-line reference for expansion and differentiation (*A1 expansion DMEM LG/differentiation DMEM LG; B3 expansion α-MEM/differentiation α-MEM*), led to a robust osteogenic differentiation compared to their corresponding negative control group, while the overall uptake of the radioactive tracer, which is proportional to the hydroxyapatite content, was higher in group A1 OSM (39,356 counts/180 s) than in group B3 (29,373 counts/180 s). However, these results were not significantly different (^99m^Tc-HDP labeling *p* = 0.485; alizarin red staining *p* = 1.0), indicating that none of these media are superior in terms of mineralization and can be assumed to be equal. As both media led to a robust osteogenic differentiation within this experiment when the same media were used for expansion and differentiation, it was expected that exchanging one for the other would not have a significant negative effect on the osteogenic differentiation (*A3: expansion DMEM LG/differentiation α-MEM; B1 expansion α-MEM/differentiation DMEM LG*). This assumption was confirmed within the results, while group A3 (*expansion DMEM LG/differentiation α-MEM*) showed an overall higher uptake of the tracer (28,948 counts) when compared to group B1 (21,572 counts, *expansion α-MEM/differentiation DMEM LG*) even though this was not statistically significant. These findings correspond with results from a previous study, where it was shown that the choice between DMEM and α-MEM for expansion of the cells does not affect osteogenic differentiation [17].

In addition, in direct comparison to the results from group A1 (*expansion DMEM LG/differentiation DMEM LG*), both media combinations A3 (*expansion DMEM LG/differentiation α-MEM*) and B1 (*expansion α-MEM/differentiation DMEM LG*) showed less uptake of the tracer and less alizarin red stain, while only a statistically lower osteogenic response was calculated for group A1 (*expansion DMEM LG/differentiation DMEM LG*) versus B1 (*expansion α-MEM/differentiation DMEM LG*) regarding the tracer uptake (*p* = 0.041). From these results, it can be assumed that the use of DMEM LG during expansion might already have exerted a significant influence on the osteogenic differentiation potential. At the same time, our data cannot support the assumption that the α-modification of the MEM medium is superior for osteogenic differentiation.

To confirm this observation, we defined group A1 (*expansion DMEM LG/differentiation DMEM LG*) as reference medium 1, as it is the gold standard for osteogenic differentiation, and tested it against all other osteogenic media combinations. As seen in the previous analysis, the “Bernese medium”, known to favor primary chondrogenic differentiation, revealed a significantly superior osteogenic differentiation for the reference group A1 compared to the two groups where “Bernese medium” was used for osteogenic differentiation (A4, *expansion group A DMEM low glucose/differentiation group 4 “Bernese medium”* and B4, *expansion group B α-MEM/differentiation group 4 “Bernese medium”*). Additionally, a significantly higher osteogenic response was shown for the reference group compared to B1 (*expansion group B α-MEM/differentiation group 1 DMEM low glucose*) and B2 (*expansion group B α-MEM/differentiation group 2 DMEM high glucose*). While these statistical significances were only observed for the results of the radioactive method, the analysis of the alizarin red staining results was only significant for the comparison of group A1 (*expansion DMEM LG/differentiation DMEM LG*) with group B4 (*expansion group B α-MEM/differentiation group 4 “Bernese medium”*). These data also substantiate the observation that DMEM LG has a valuable impact on osteogenic differentiation. A possible explanation regarding the discrepancy of significance between the radioactive method and the alizarin red staining method is that the radioactive labeling method is more sensitive for the detection of mineralization in vitro. Surprisingly, no statistical significance was shown for a superiority of the “Verfaillie medium”, which is known within the literature to boost osteogenic differentiation [29], yet there was a trend towards a higher uptake for this group (*A5 expansion group A DMEM low glucose/differentiation group 5 “Verfaillie medium”*).

Unlike the prior observations, by defining group B3 as the second reference group (*expansion group B α-MEM/differentiation group 3 α-MEM*) for only the direct comparison with group A4 (*expansion group A DMEM low glucose/differentiation group 4 “Bernese medium”*) and B4 (*expansion group B α-MEM/differentiation group 4 “Bernese medium”*), a significant superiority of the reference medium was calculated, probably due to the same reasons as already mentioned. Towards all other media combinations, the secondary reference medium did not lead to a stronger osteogenic response.

These results might be a hint that the general usage of DMEM LG for cell expansion has a notable effect on the overall osteogenic response. In addition, we performed a Mann–Whitney U test comparing all groups that were previously expanded in DMEM LG with all groups that were previously expanded in α-MEM. Even if the overall uptake of the tracer and alizarin red stain was higher when previously expanded in DMEM LG (29,700 counts/3.27 µg/mL) than in α-MEM (22,033 counts/2.87 µg/mL), no statistical significance was shown (*p* = 0.287 and *p* = 0.865). To further investigate if there were any significant differences regarding the media used for expansion, pairs were formed of the groups where a similar media was used for differentiation while the expansion media was either α-MEM or DMEM LG. Here, only the comparison of group A1 (*expansion DMEM LG/differentiation DMEM LG*) with B1 (*expansion α-MEM/differentiation DMEM LG*) revealed a significant effect for the radioactive labeling method (*p* = 0.041) while the alizarin red staining showed no significance (*p* = 1.0). All other pairs showed no significant difference, which can also lead to the assumption that the positive impact of DMEM LG upon osteogenic differentiation already starts during cell expansion. These data concur with findings from other groups that investigated the importance of expansion media during osteogenic differentiation, as the expansion media seems to determine the stem cell character for future usage [18].

During the study, the general observation was made that statistical differences between some groups were only proven by ^99m^Tc-HDP labeling, while the previous gold-standard alizarin red staining showed no significant difference at all. Only for the direct comparison of the group A4 OSM (*expansion group A DMEM low glucose/differentiation group 4 “Bernese medium”*) with the corresponding negative control group A4 CNTRL (*expansion group A DMEM low glucose/differentiation group 4 “Bernese medium”*) did the Mann–Whitney U test reveal a significant difference. A deeper analysis of the data resulted in the observation that statistics, made with the results from the ^99m^Tc-HDP labeling, significantly confirmed the observed trends. This is a proof of earlier results which have already indicated that the method of ^99m^Tc-HDP labeling is highly significant and sensitive for osteogenic quantification and for revealing very exact differences [34]. Hence, it was no surprise that the correlation analysis of the ^99m^Tc-HDP labeling with the alizarin red staining showed a highly significant correlation (2-sided significance *p* ≤ 0.001) with a very robust Pearson correlation coefficient of r^2^ = 0.799.

Within our study, only mesenchymal stromal (stem) cells from the proximal femoral bone were used. Therefore, our results are limited to this specific cell population, while it remains unclear whether these results could be transferred to an MSC population harvested from other sites (e.g., iliac crest). There is evidence that the phenotype and differentiation potential of MSCs is equal in the iliac crest as in long bones, which might be a hint that the results could be similar [39]. This must be investigated in a subsequent experimental setup. In addition, certain environmental impacts (e.g., trauma, chronic infections) on the MSCs before their harvest can result in a “memory” effect which has an influence on their proliferation and differentiation abilities [40]. Therefore, our media recommendations should be critically evaluated before being applied to a specific cell culture and shouldn’t be generalized.

Our study deliberately only included the evaluation of cell proliferation in the different media during the expansion phase, due to the focus upon synthesis of a mineralizing matrix during the differentiation period. Therefore, within the experimental setup all cultures were standardized to an expansion period of 10 days, followed by an osteogenic differentiation for 21 days, while the cell number was equally normalized prior to the transfer to the 35 mm dishes in which the osteogenic differentiation was performed. All cell cultures showed >99% confluence by the end of the experiment.

Furthermore, within this study the key focus was the mineralization response of the MSCs under various cell culture media combinations, as this is one of the most important factors in bone regeneration. It might be possible that an evaluation of other key markers (e.g., alkaline phosphatase) or gene expression levels would reveal deviating results. Beside the hydroxyapatite deposition of osteogenic differentiation of BM-MSCs, cell migration and cell-cell interactions as well as paracrine and autocrine secretion are also of high importance in complex in vitro models to mimic possible scenarios in vivo.

In a follow-up study, we will investigate whether there is any impact upon osteogenic differentiation if more elaborate media, such as “Bernese medium” or “Verfaillie medium” are used for cell expansion followed by osteogenic differentiation. Further investigations are planned within this study to evaluate the elementary structure of the synthesized hydroxyapatite as well as the osteogenic key markers and cell migration and cell-cell interactions.

## 4. Materials and Methods

### 4.1. Experimental Design at a Glance

Human mesenchymal stem cells were harvested from the femoral bone cavity of healthy donors (*n* = 6). Then, 250,000 cells from each donor were seeded into T-150 flat bottom flasks in duplicates. While one of the flasks of each donor received DMEM low glucose as basal cell culture media (expansion group A) for cell expansion, the other flask received α-MEM (expansion group B). After 10 days, cells from every donor and expansion group were transferred into ten 35 mm diameter petri dishes. Dishes 1–5 were differentiated towards the osteogenic lineage using the standard osteogenic supplements (OSM) dexamethasone, ascorbic acid, and ß-glycerol phosphate, together with DMEM low glucose (dish 1, differentiation group 1), DMEM high glucose (dish 2, differentiation group 2), α-MEM (dish 3, differentiation group 3), “Bernese medium” (dish 4, differentiation group 4), “Verfaillie medium” (dish 5, differentiation group 5), while dishes 6–10 received the same cell culture media but without the osteogenic supplements, and served as negative control groups (CNTRL): DMEM low glucose (dish 6, control group 1), DMEM high glucose (dish 7, control group 2), α-MEM (dish 8, control group 3), “Bernese medium” (dish 9, control group 4), “Verfaillie medium” (dish 10, control group 10). This setup led to 20 different groups in total, where 10 groups served as osteogenic differentiation groups and 10 groups were negative control groups. Figure 1 gives a detailed overview of all groups.

(1) Group A1 OSM: expansion group A DMEM low glucose/differentiation group 1 DMEM low glucose with osteogenic supplements (OSM) + 10% FCS and 1/Pen/Strep.
(2) Group A1 CNTRL: expansion group A DMEM low glucose/control group 1 DMEM low glucose without osteogenic supplements (CNTRL) + 10% FCS and 1/Pen/Strep.
(3) Group A2 OSM: expansion group A DMEM low glucose/differentiation group 2 DMEM high glucose with osteogenic supplements (OSM) + 10% FCS and 1/Pen/Strep.
(4) Group A2 CNTRL: expansion group A DMEM low glucose/control group 2 DMEM high glucose without osteogenic supplements (CNTRL) + 10% FCS and 1/Pen/Strep.
(5) Group A3 OSM: expansion group A DMEM low glucose/differentiation group 3 α-MEM with osteogenic supplements (OSM) + 10% FCS and 1/Pen/Strep.
(6) Group A3 CNTRL: expansion group A DMEM low glucose/control group 3 α-MEM without osteogenic supplements (CNTRL) + 10% FCS and 1/Pen/Strep.
(7) Group A4 OSM: expansion group A DMEM low glucose/differentiation group 4 “Bernese medium” with osteogenic supplements (OSM) + 10% FCS and 1/Pen/Strep.
(8) Group A4 CNTRL: expansion group A DMEM low glucose/control group 4 “Bernese medium” without osteogenic supplements (CNTRL) + 10% FCS and 1/Pen/Strep.
(9) Group A5 OSM: expansion group A DMEM low glucose/differentiation group 5 “Verfaillie medium” with osteogenic supplements (OSM) + 10% FCS and 1/Pen/Strep.
(10) Group A5 CNTRL: expansion group A DMEM low glucose/control group 5 “Verfaillie medium” without osteogenic supplements (CNTRL) + 10% FCS and 1/Pen/Strep.
(11) Group B1 OSM: expansion group B α-MEM/differentiation group 1 DMEM low glucose with osteogenic supplements (OSM) + 10% FCS and 1/Pen/Strep.
(12) Group B1 CNTRL: expansion group B α-MEM/control group 1 DMEM low glucose without osteogenic supplements (CNTRL) + 10% FCS and 1/Pen/Strep.
(13) Group B2 OSM: expansion group B α-MEM/differentiation group 2 DMEM high glucose with osteogenic supplements (OSM) + 10% FCS and 1/Pen/Strep.
(14) Group B2 CNTRL: expansion group B α-MEM/control group 2 DMEM high glucose without osteogenic supplements (CNTRL) + 10% FCS and 1/Pen/Strep.
(15) Group B3 OSM: expansion group B α-MEM/differentiation group 3 α-MEM with osteogenic supplements (OSM) + 10% FCS and 1/Pen/Strep.
(16) Group B3 CNTRL: expansion group B α-MEM/control group 3 α-MEM without osteogenic supplements (CNTRL) + 10% FCS and 1/Pen/Strep.
(17) Group B4 OSM: expansion group B α-MEM/differentiation group 4 “Bernese medium” with osteogenic supplements (OSM) + 10% FCS and 1/Pen/Strep.
(18) Group B4 CNTRL: expansion group B α-MEM/control group 4 “Bernese medium” without osteogenic supplements (CNTRL) + 10% FCS and 1/Pen/Strep.
(19) Group B5 OSM: expansion group B α-MEM/differentiation group 5 “Verfaillie medium” with osteogenic supplements (OSM) + 10% FCS and 1/Pen/Strep.
(20) Group B5 CNTRL: expansion group B α-MEM/control group 5 “Verfaillie medium” without osteogenic supplements (CNTRL) + 10% FCS and 1/Pen/Strep.

After 21 days, the cell cultures were terminated, and the hydroxyapatite content was accessed using ^99m^Tc-HDP labeling. As this technique is non-destructive, the amount of hydroxyapatite was subsequently verified by quantitative alizarin red staining.

### 4.2. hMSC Harvest and FACS Analysis

Bone marrow aspirates were obtained from the proximal femoral cavity of six healthy donors (*n* = 6) under general anesthesia during an elective surgical procedure for total hip arthroplasty after informed consent. During the preparation of the proximal femoral bone cavity, 10 mL of bone marrow was collected in a 20 mL syringe (BD) containing 1000 IU of heparin (Braun). Individual samples were diluted 1:1 with PBS (Invitrogen, Waltham MA, USA) and washed twice with PBS. The mononuclear cell fraction was isolated by Ficoll gradient centrifugation (Ficoll-Paque-PLUS; GE Healthcare). Mononuclear cells were plated in T-150 polystyrene tissue culture flasks (Falcon, BD, Heidelberg, Germany) at a density of 500,000/cm^2^ and cultured in a humidified 5% CO_2_ atmosphere at 37 °C in low-glucose DMEM LG (Invitrogen) containing 10% heat-inactivated (56 °C, 30 min) fetal bovine serum (Invitrogen) and 1% Penicillin/Streptomycin (Invitrogen). After 48 h, nonadherent cells were removed by washing with PBS, while the adherent cells were defined as bone marrow mesenchymal stem cells. The medium was changed every 2–3 days. At 90% of confluence, cells were trypsinized.

For further experiments, these P0 cells were frozen in liquid nitrogen in 0.5 mL aliquots containing 500,000 cells with 10% DMSO (Sigma).

To ensure that the harvested cells were indeed mesenchymal stromal cells, they were subsequently analyzed by FACS analysis regarding their specific surface antigen expression (for CD 105+, CD 73+, CD 90+, CD45-, CD 34-, CD 14/CD112b-, CD79a/CD19-, HLA-DR-) using a flow cytometer (Miltenyi Biotec, MACSQuant SN 2173) and subsequent analyzation (MACSQuantify FlowJo 9.8.1.). The phenotype criteria were defined as stated by the International Society for Cellular Therapy [36].

### 4.3. hMSC Expansion

P0 human MSCs (*n* = 6) were thawed and seeded in duplicates into a T-150 flask (Falcon) with 250,000 cells each, and cultured for 10 days in a humidified 5% CO_2_ atmosphere at 37 °C. The medium was changed every 2–3 days. During this cell expansion stage, one flask of each donor received DMEM low glucose + 10% FCS and 1% Pen/Strep (expansion group A) as expansion medium, while the other flask of the same donor received α-MEM + 10% FCS and 1% Pen/Strep (expansion group B). After 10 days, 80–90% confluence was reached in all cell cultures, and cells were trypsinized, counted, and resuspended. The 10-day expansion period was chosen to ensure that all cells had been exposed to the corresponding media for the same amount of time.

### 4.4. hMSC Differentiation

Cells from every donor (*n* = 6) and expansion group (A and B) were then seeded ten-fold at a density of 15,000 cells/cm^2^ into 35 mm flat bottom petri dishes (Corning), leading to a total of 20 dishes per donor. Each of the dishes received one of the five differentiation media: DMEM low glucose, DMEM high glucose, α-MEM, “Bernese medium”, or “Verfaillie medium”, with (dish 1–5) or without (dish 6–10) osteogenic supplements (see Section 4.1).

Cells were treated for 21 days, with medium change every 2–3 days and cultured in a humidified 5% CO_2_ atmosphere at 37 °C. After 21 days, the cell cultures were terminated and washed twice with PBD, followed by air drying.

### 4.5. ^99m^Tc- HDP Labeling and Analysis

Each dish had 5.5 MBq ^99m^Tc-HDP in 2 mL of NaCl 0.9% added to it. The radioactivity was assayed with a dose calibrator (Activimeter ISOMED 1010, Nuklear-Medizintechnik Dresden GmbH, Dresden, Germany). The dishes were then incubated at room temperature for 2 h. The remaining liquid ^99m^Tc-HDP was removed, and the dishes were washed three times (for approximately 30 min) in PBS to remove the unbound radiotracer. Then, the dishes were placed under a gamma camera (E.CAM+, Siemens, Erlangen, Germany) directly on the lower detector, and the counts were acquired using this detector. The radionuclide counts were acquired for 180 s, and then the images were analyzed using Xeleris Software (GE Healthcare).

The total number of counts for each culture dish was determined by placing a circular region of interest (ROI) around each dish. All ROIs had the same pixel size and were chosen to be a bit smaller than the true diameter of the dish, so that the very outer parts were excluded to avoid partial volume effects. Then the total gamma counts emitted from each ROI were measured over 180 s.

One blank dish was labeled in parallel each time to detect the amount of background radionuclide binding. The amount of the blank binding was subtracted from the results obtained from the dishes containing cells.

### 4.6. Quantitative Alizarin Red Staining

For mineral semi-quantification of the monolayer cell cultures, the dishes previously investigated by ^99m^Tc-HDP labeling were used. The 35 mm dishes were fixed with 2.5 mL 70% ethanol (−20 °C) for 4 min, followed by washing with aqua dest. For the preparation of the staining solution, 0.5 g Alizarin-Red S powder was dissolved in 100 mL aqua dest (Sigma-Aldrich A5533, St. Louis, MO, USA). The pH was measured using a pH meter and by adding ammonium hydroxide (Sigma 221228, St. Louis, MO, USA). The pH was adjusted to 7.2. Subsequently, 1 ml of the staining solution was added to each dish, and these were incubated at room temperature for 10 min followed by 5× washing with 1 ml aqua dest. Then 1 mL of a 10% (*w*/*v*) cetylpyridinium chloride solution dissolved in 10 mM sodium phosphate was added to each dish. After incubation for 10 min at room temperature on a shaker, 20 µL from each sample was put in a 96-well plate, and 180 µL of the 10% (*w*/*v*) cetylpyridinium chloride solution was added.

In parallel, standard dilution with an alizarin red concentration of between 62.5 µg/mL and 0.01 µg/mL was prepared in the 96-well plate.

Photometric absorption was determined at 570 nm in a 96-well plate reader, followed by plotting a standard curve with the results of the standard dilution. The absorption values of the samples were transferred into alizarin red concentration by referring to the standard curve.

### 4.7. Statistics and Evaluation

All results were tested for normal distribution using the Kolmogorov–Smirnov test before further tests were performed. Subsequently, the global Kruskal–Wallis test was performed to test for general statistical significances between the groups, followed by Mann–Whitney U tests to determine differences regarding tracer uptake and alizarin red concentration between the osteogenic group (OSM) and the corresponding negative control group (CNTRL). These tests were used due to the small individual sample size of each group (*n* = 6).

The Mann–Whitney U test was also used to test for statistical differences between the ten osteogenic groups. Here, group A1 (OSM: expansion group A DMEM low glucose/differentiation group 1 DMEM low glucose with osteogenic supplements), and group B3 (OSM: expansion group B α-MEM/differentiation group 3 α-MEM with osteogenic supplements) were defined as reference groups.

For validation of the ^99m^Tc-HDP labeling, the results were correlated with the results from the quantitative alizarin red staining using Pearson’s correlation coefficient.

Statistical analyses were performed using SPSS Statistics^®^ (IBM) Version 27. Statistical significance was set to *p* ≤ 0.05.

As this study generated a large amount of data, a 7-step procedure was defined prior to analyzing the results to ensure a structured evaluation of the data and avoid multiple testing.

Step 1: Descriptive analysis of all 20 groups.

Step 2: Kolmogorov–Smirnov test for normal distribution of all 20 groups followed by Kruskal–Wallis test for global significance.

Step 3: Mann–Whitney U test for all osteogenic groups (OSM) and the corresponding negative control group (CNTRL) to evaluate if any significant ossification had occurred.

Step 4: As DMEM LG reflects the gold standard for osteogenic differentiation, group A1 OSM (expansion DMEM LG/differentiation DMEM LG) was defined as the reference group and a Mann–Whitney U test for all osteogenic groups (OSM) was performed to investigate whether any of the other osteogenic media combinations were superior to the gold standard.

Step 5: As α-MEM is also very common for expansion of MSCs followed by osteogenic differentiation, group B3 (expansion α-MEM/differentiation α-MEM) was defined within a second dataset as the secondary reference group. Then all other osteogenic groups were tested, using a Mann–Whitney U test, against the secondary reference media.

Step 6: To evaluate if there was any significant difference regarding the osteogenic response if the expansion media was either DMEM LG or α-MEM while the same media were used for differentiation, a Mann–Whitney U test for the corresponding pairs for the expansion media was performed for all pairs.

Step 7: Finally, to validate the ^99m^Tc-HDP labeling, a Pearson correlation was performed with the gold-standard alizarin red staining.

## 5. Conclusions

This research article investigated multiple media combinations to verify if any of these media were superior for boosting osteogenic differentiation. The following main conclusions can be drawn from the results:

1. All cell culture media used led to a robust osteogenic differentiation and can, therefore, be used to prove the differentiation into the osteogenic lineage with the exception of the “Bernese medium”. This not at all suitable for promoting osteogenic differentiation regardless of whether the cells have been expanded in DMEM LG or α-MEM.
2. “Verfaillie medium”, used for differentiation after expansion in DMEM LG, led to the highest osteogenic response yet did not significantly differ from the reference medium DMEM LG for expansion and differentiation.
3. DMEM LG used for expansion and differentiation is superior regarding the osteogenic response to α-MEM expansion followed by DMEM LG differentiation, as well as α-MEM expansion followed by DMEM HG differentiation.
4. DMEM LG is overall the most suitable expansion and differentiation medium for the osteogenic lineage as it led to a robust osteogenic response (second highest counts after “Verfaillie medium”) while it is much more economical in terms of cost and time required. Therefore, we recommend DMEM LG for cell expansion and cell differentiation when investigating the osteogenic lineage differentiation of MSCs.

## Figures and Tables

**Table 1:** Paired Mann–Whitney U Test for each osteogenic group (OSM) and the corresponding negative control group (CNTRL). Significant results are indicated by ** *p* ≤ 0.01; * *p* ≤ 0.05.

Group	^99m^Tc-HDP Labeling Mean Gamma Counts/180 s	Alizarin Red Staining Concentration µg/mL	^99m^Tc-HDP Labeling Exact Significance (*p*-Value)	Alizarin Red Staining Exact Significance (*p*-Value)
**A1 OSM; DMEM LG/DMEM LG**	39,356	3.01	0.002 **	0.004 **
**A1 CNTRL; DMEM LG/DMEM LG**	3806	0.51		
**A2 OSM; DMEM LG/DMEM HG**	30,846	2.54	0.002 **	0.002 **
**A2 CNTRL; DMEM LG/DMEM HG**	4981	0.42		
**A3 OSM; DMEM LG/** **α** **-MEM**	28,948	4.36	0.004 **	0.002 **
**A3 CNTRL; DMEM LG/** **α** **-MEM**	2945	0.33		
**A4 OSM; DMEM LG/BERNESE**	2025	1.90	0.132	0.026 *
**A4 CNTRL: DMEM LG/BERNESE**	3381	0.48		
**A5 OSM; DMEM LG/VERF**	47,328	4.54	0.026 *	0.04 *
**A5 CNTRL; DMEM_LG/VERF**	3315	0.43		
**B1 OSM; α** **-MEM/DMEM LG**	21,572	2.81	0.002 **	0.002 **
**B1 CNTRL; α** **-MEM/DMEM LG**	2574	0.44		
**B2 OSM; α** **-MEM/DMEM HG**	17,382	3.07	0.002 **	0.002 **
**B2 CNTRL: α** **-MEM/DMEM HG**	2820	0.42		
**B3 OSM; α** **-MEM/** **α** **-MEM**	29,373	3.06	0.015 *	0.004 **
**B3 CNTRL; α** **-MEM/** **α** **-MEM**	2783	0.50		
**B4 OSM; α** **-MEM/BERNESE**	2166	0.86	0.310	1.000
**B4 CNTRL; α** **-MEM/BERNESE**	3004	0.62		
**B5 OSM; α** **-MEM/VERF**	39,670	4.54	0.015 *	0.009 **
**B5 CNTRL; α** **-MEM/VERF**	3078	0.63		

**Table 2:** Mann–Whitney Test for all osteogenic groups (OSM) when group A1 OSM is defined as the reference group. Significant results are in bold and indicated by ** *p* ≤ 0.01; * *p* ≤ 0.05.

Reference Group	Reference Gamma Counts/180 s	Reference Alizarin Red Concentration µg/mL	Test Group	Test Group Gamma Counts/ 180 s	Test Group Alizarin Red Concentration µg/mL	^99m^Tc-HDP Labeling Exact Significance (*p*-Value)	Alizarin Red Staining Exact Significance (*p*-Value)
**A1 OSM; DMEM LG/DMEM LG**	39,356	3.01	A2 OSM; DMEM LG/DMEM HG	30,846	2.54	0.394	0.699
			A3 OSM; DMEM LG/α-MEM	28,948	4.36	0.394	1.000
			A4 OSM; DMEM LG/BERNESE	2025	1.90	0.002 **	0.093
			A5 OSM; DMEM LG/VERF	47,328	4.54	0.240	0.310
			B1 OSM; α-MEM/DMEM LG	21,572	2.81	0.041 *	1.000
			B2 OSM; α-MEM/DMEM HG	17,382	3.07	0.026 **	0.937
			B3 OSM; α-MEM/α-MEM	29,373	3.06	0.485	1.000
			B4 OSM; α-MEM/BERNESE	2166	0.86	0.002 **	0.015 **
			B5 OSM; α-MEM/VERF	39,670	4.54	0.699	0.485

**Table 3:** Mann–Whitney Test for all osteogenic groups (OSM) when group B3 OSM is defined as the reference group. Significant results are indicated by ** *p* ≤ 0.01; * *p* ≤ 0.05.

Reference Group	Reference Gamma Counts/180 s	Reference Alizarin Red Concentration µg/mL	Test Group	Test Group Gamma Counts/ 180 s	Test Group Alizarin Red Concentration µg/mL	^99m^Tc-HDP Labeling Exact Significance (*p*-Value)	Alizarin Red Staining Exact Significance (*p*-Value)
B3 OSM; α-MEM/α-MEM	29,373	3.06	A1 OSM; DMEM LG/DMEM LG	36,356	3.01	0.485	1.000
			A2 OSM; DMEM LG/DMEM HG	30,846	2.54	0.818	0.485
			A3 OSM; DMEM LG/α-MEM	28,948	4.36	0.937	0.937
			A4 OSM; DMEM LG/BERNESE	2025	1.90	0.015 *	0.240
			A5 OSM; DMEM LG/VERF	47,328	4.54	0.180	0.240
			B1 OSM; α-MEM/DMEM LG	21,572	2.81	0.485	0.699
			B2 OSM; α-MEM/DMEM HG	17,382	3.07	0.485	0.937
			B4 OSM; α-MEM/BERNESE	2166	0.86	0.009 **	0.065
			B5 OSM; α-MEM/VERF	39,670	4.54	0.310	0.485
